# Mr. Whately, on Ulcerations in the Urethra

**Published:** 1805-04-01

**Authors:** Thomas Whately

**Affiliations:** Grafton Street


### *To the Editors of the Medical and Physical Journal.*

GENTLEMEN,

ON looking over your Journal for January last, I was astonished at seeing the following paragraph in your Review of Mr. Platt's "Inquiry into the Efficacy of Oxygen in the cure of Syphilis."

——"Mr. Whately's revived notion of ulcers in the urethra is introduced; and Mr. Platt expresses his surprise at an opinion so inconsistent with experience, an accurate knowledge of the parts, and of the disease." Medical and Physical Journal, No. 71. p. 79. I shall now give a *faithful extract* of Mr. Platt's own words, from whence this quotation is taken.——"It is with some degree of surprise, I observed in a very recent publication,* a revival of those opinions which have been considered almost obsolete. It is *there* remarked, that gonorrhœa is *invariably attended with ulcerations in the urethra.* Various authorities are quoted by this gentleman in support of the accuracy of his opinion, but most of those authorities are of a date anterior to the period when any difference on this subject prevailed, &c."†—— I shall next give a *faithful copy* of nearly the whole of page 12 of my Essay on Gonorrhœa, from which this gentleman has made his extract, and expressed so much *surprise.*

"It has been believed, that the gonorrhœa arose most frequently from ulcerations in different parts of the urethra, or the glands adjoining to it; but the researches of modern anatomists seem very nearly to have overthrown this opinion; and it has of late been asserted, that an ulceration of the urethra never takes place in this disease. This doctrine appears to have been first advanced about the year 1750, by that celebrated anatomist the late Dr. William Hunter, who taught it in his lectures. But although it is certainly true, that in *most* instances of gonorrhœa there is *no ulceration* of the urethra; it is equally certain that this is not universally the case, as such a disease does *sometimes, though rarely,* occur. In my own practice, I have in a common gonorrhœa, *now and then,* met with ulcers in the urethra. Where its orifice has been naturally wide, I have *several times* distinctly seen them,

---

* Whately's Practical Observations on the Gonorrhœa Virulenta, p. 12.

† See Platt's Inquiry into the Efficacy of Oxygen, &c. p. 19.

both a little within, and at the extremity of this canal. In a *few other cases,* I have had no doubt but that ulcers have existed in the urethra, &c." p. 12.

After these extracts it might be deemed unnecessary to add any remarks; but as Mr. Platt may possibly refer to other parts of my work in support of his assertions, and as my expressions on the difference in excoriation on the surface of the membrane of the urethra in the different species of gonorrhœa may be misconstrued into evident ulcerations of that membrane, if not taken in connection with other sentences, I shall make a few other extracts, in order to prevent a controversy on this business.—"Where neither of the kind of ulcers before-mentioned exist in the urethra, there is certainly a considerable difference discernable in the depth to which the poison penetrates into or excoriates the inner membrane of the urethra in different cases of gonorrhœa, &c. &c."—— In a note at this place I have said, "That in some of these cases, this disease is a mere superficial inflammation of the inner membrane of the urethra, without any apparent irregularities on its surface, or any induration of the corpus spongiosum; in others, the diseased surface is unequal and rough, like that of a file, and when minutely viewed, would, no doubt, be found to be a congeries of ulcerations. I have seen such a surface so raw as to bleed after every evacuation of the urine." Ib. page 17.—In a subsequent part of this Essay, I have divided this disease into three different species.

1st. "The gonorrhœa attended with ulcer in the urethra, &c."

2d. "The gonorrhœa attended with marks of a considerable inflammatory affection of the urethra, *but without any appearance of ulcer.*"

3d. "The gonorrhœa *unattended with any of these circumstances,* its *chief* symptom being a small purulent discharge from the urethra." p. 33.

In the following page I have remarked, that in all gonorrhœas of the second species, "We have reason to conclude from the observations already made, that the poison has penetrated into and excoriated the inner membrane of the urethra to some depth, though it *may not have produced actual ulcers in it.*" p. 34.

I have made these extracts as concisely as possible, that I might not occupy too much room in your valuable Journal.—I shall enter into no controversy on the subject, but conclude with expressing *my surprise,* that any gentleman should should be guilty of misrepresenting the meaning of any author from whom he differs in opinion.

I am, &c.

*Grafton Street, Feb.* 15, 1805. THOMAS WHATELY.

---

